# Trib2 Suppresses Tumor Initiation in Notch-Driven T-ALL

**DOI:** 10.1371/journal.pone.0155408

**Published:** 2016-05-18

**Authors:** Sarah J. Stein, Ethan A. Mack, Kelly S. Rome, Kostandin V. Pajcini, Takuya Ohtani, Lanwei Xu, Yunlei Li, Jules P. P. Meijerink, Robert B. Faryabi, Warren S. Pear

**Affiliations:** 1 Department of Pathology and Laboratory Medicine, Abramson Family Cancer Research Institute, Institute of Medicine and Engineering, Institute for Immunology, Center for Personalized Diagnostics, Perelman School of Medicine at the University of Pennsylvania, Philadelphia, PA, United States of America; 2 Department of Pharmacology, University of Illinois at Chicago, Chicago, IL, United States of America; 3 The Department of Pediatric Oncology/Hematology, Erasmus Medical Center, Rotterdam, the Netherlands; B.C. Cancer Agency, CANADA

## Abstract

Trib2 is highly expressed in human T cell acute lymphoblastic leukemia (T-ALL) and is a direct transcriptional target of the oncogenic drivers Notch and TAL1. In human TAL1-driven T-ALL cell lines, Trib2 is proposed to function as an important survival factor, but there is limited information about the role of Trib2 in primary T-ALL. In this study, we investigated the role of Trib2 in the initiation and maintenance of Notch-dependent T-ALL. Trib2 had no effect on the growth and survival of murine T-ALL cell lines *in vitro* when expression was blocked by shRNAs. To test the function of Trib2 on leukemogenesis *in vivo*, we generated Trib2 knockout mice. Mice were born at the expected Mendelian frequencies without gross developmental anomalies. Adult mice did not develop pathology or shortened survival, and hematopoiesis, including T cell development, was unperturbed. Using a retroviral model of Notch-induced T-ALL, deletion of *Trib2* unexpectedly decreased the latency and increased the penetrance of T-ALL development *in vivo*. Immunoblotting of primary murine T-ALL cells showed that the absence of *Trib2* increased C/EBPα expression, a known regulator of cell proliferation, and did not alter AKT or ERK phosphorylation. Although Trib2 was suggested to be highly expressed in T-ALL, transcriptomic analysis of two independent T-ALL cohorts showed that low Trib2 expression correlated with the TLX1-expressing cortical mature T-ALL subtype, whereas high Trib2 expression correlated with the LYL1-expressing early immature T-ALL subtype. These data indicate that Trib2 has a complex role in the pathogenesis of Notch-driven T-ALL, which may vary between different T-ALL subtypes.

## Introduction

T cell acute lymphoblastic leukemia (T-ALL) is an aggressive hematologic malignancy resulting from the oncogenic transformation of T cell progenitors and accounting for 25% of adult and 15% of pediatric ALL cases [1]. Although intensified chemotherapy has markedly improved long-term survival, survival following relapse is poor, highlighting the need for novel therapies in recurrent disease. Activating mutations of Notch1 occur in about 50% of T-ALL cases, most of which occur in the heterodimerization domain (HD) and proline-glutamic acid- serine-threonine (PEST) domains [2]. NOTCH1 is a potentially important therapeutic target in T-ALL given the high prevalence of mutations and the significant role of NOTCH signaling in T-ALL. Although inhibiting Notch in mouse models of T-ALL caused marked anti-leukemic effects *in vivo* [3, 4], success in patients has met with difficulties, in part due to “on target” toxicity and possible drug resistance [5]. Therefore, identifying downstream Notch effectors may lead to the identification of novel therapeutic targets in T-ALL.

Previous studies identified Trib2, a member of the Tribbles protein family, as a direct transcriptional target of Notch1 in T-ALL cell lines [6, 7]. Tribbles are an evolutionarily conserved protein family that is implicated in diverse cellular events that include proliferation, migration, metabolism, and oncogenic transformation (reviewed in [8]). Tribbles was first characterized in *Drosophila* as an important cell cycle regulator [9]. In mammals, the Trib protein family members Trib1, Trib2 and Trib3 are characterized by a conserved pseudokinase domain [10], and COP1 [11] and MEK1 [12] binding domains. Trib proteins function as scaffolding molecules that facilitate protein degradation via a proteasome-dependent mechanism. In mammals, Trib1 and Trib2 promote C/EBPα degradation [6, 13] by recruiting the E3 ligase, COP1. Similarly, Trib3 promotes COP1-dependent degradation of acetyl CoA carboxylase (ACC), an enzyme involved in fatty acid synthesis [11]. The Trib proteins also modulate signaling pathways such as AKT [14] and MAPK [15]. Trib2 can inhibit AKT phosphorylation [16, 17] and Trib proteins interact with MEK1 and enhance ERK phosphorylation [15] through the MEK1 binding motif [12].

Trib2 is highly expressed in human T-ALL and T cells [18]. In addition to its association with Notch expression in T-ALL, a recent study showed that Trib2 is required for the growth and survival of human T-ALL cell lines driven by TAL1 [19]. In contrast, we found that shRNA-mediated knockdown of Trib2 in murine T-ALL cell lines did not affect cell growth or survival. To investigate the importance of Trib2 in T cell development and in T-ALL pathogenesis *in vivo*, we generated Trib2 knockout mice. Our results show that global deletion of Trib2 had no obvious defects on mouse development or survival. In particular, T cell development was normal. Strikingly, the absence of Trib2 resulted in a significant decrease in disease latency and increased disease penetrance in a murine model of Notch-driven T-ALL. Tumor cells from moribund mice expressed increased C/EBPα protein, while no change was seen in the amounts of phosphorylated AKT or ERK. Taken together, these data indicate that the influence of Trib2 expression on T cell leukemogenesis may vary in different contexts; however, in murine T-ALL, we identified an important role for Trib2 as a negative regulator of Notch-induced T-ALL *in vivo*.

## Results

### Murine T-ALL cells do not depend on persistent Trib2 expression

**Fig 1:**
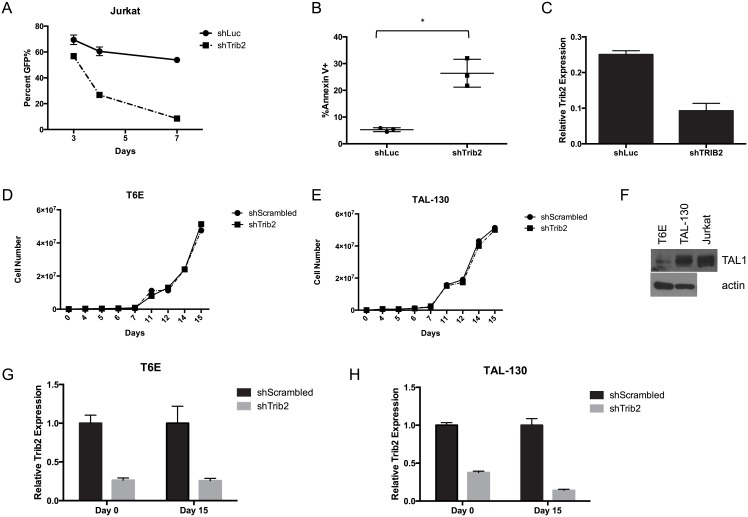
Trib2 is not required to maintain murine T-ALL cell lines. Jurkat cells were transduced with normalized viral particles to express shRNAs against Luciferase (shLuc) or Trib2 (shTrib2) along with a GFP reporter. A) The percentage of GFP^+^ cells within the population was monitored for 7 days post-infection. B) At 3 days post-transduction, apoptosis was measured by Annexin V staining (“*”, p = 0.018) and C) Trib2 expression was measured by qPCR 3 days post-transduction in sorted GFP^+^ cells. Errors bars represent the standard deviation of biological replicates. D) T6E cells were transduced with shRNAs against a scrambled sequence (shScrambled) or Trib2 (shTrib2) and GFP as a surrogate marker. GFP^+^ cells were sorted 48 hours after transduction (day 0) and growth was monitored. E) TAL-130 cells were transduced with shRNAs against a scrambled sequence (shScrambled) or Trib2 (shTrib2) and GFP as a surrogate marker. GFP^+^ cells were sorted 48 hours after transduction (day 0) and growth was monitored. F) TAL1 expression was measured in T6E and TAL-130 cells by immunoblot. Jurkat lysates were used as a positive control. G & H) Trib2 expression was measured at days 0 and 15 in T6E (Panel G) or TAL-130 cells (Panel H). Error bars indicate standard deviation. Data are representative of 3 experiments.

In order to determine the importance of Trib2 in the maintenance of T-ALL, we investigated the effect of Trib2 loss in T-ALL cell lines. A previous study identified Trib2 as an important survival factor in human TAL1-driven T-ALL cell lines, including Jurkat [19]. Consistent with these data, we observed a severe decrease in Jurkat cell growth (Fig 1A) due to apoptosis (Fig 1B) when Trib2 was knocked down (Fig 1C), suggesting an important role for Trib2 in the growth/survival of these cells. Activating mutations in NOTCH1 occur in over 50% of human T-ALLs [2]. Although Notch1 is mutated in Jurkat cells, growth and survival of these cells is Notch-independent [20, 21]. As *Trib2* is a direct Notch target in Notch-induced murine T-ALL cells [7], we investigated the requirement for persistent Trib2 expression in these Notch-dependent cells. T6E cells [22], a murine Notch1-dependent T-ALL cell line, were transduced with shRNAs targeting *Trib2* or a scrambled control sequence. Unlike Jurkat cells, *Trib2* knockdown did not affect cell growth or survival (Fig 1D and data not shown). To determine if Trib2 dependency is specific for Tal1 expressing cell lines, we assayed the effects of Trib2 knockdown in TAL-130 cells, a T-ALL cell line derived from mice expressing a Tal1 transgene [23]. Knockdown of Trib2 by shRNA in TAL-130 cells showed no changes in cell growth (Fig 1E) or survival (data not shown). Immunoblotting confirmed that TAL-130 cells express higher levels of Tal1 than T6E cells (Fig 1F). Furthermore, both T6E and TAL-130 cells maintained Trib2 knockdown for at least 15 days after expressing the shRNA (Fig 1G and 1H), indicating that neither cell line requires Trib2 for proliferation or survival. Together, these data indicate that the requirement for persistent Trib2 expression varies between different T-ALL cell lines.

### Absence of T cell defects in Trib2 knockout mice

**Fig 2:**
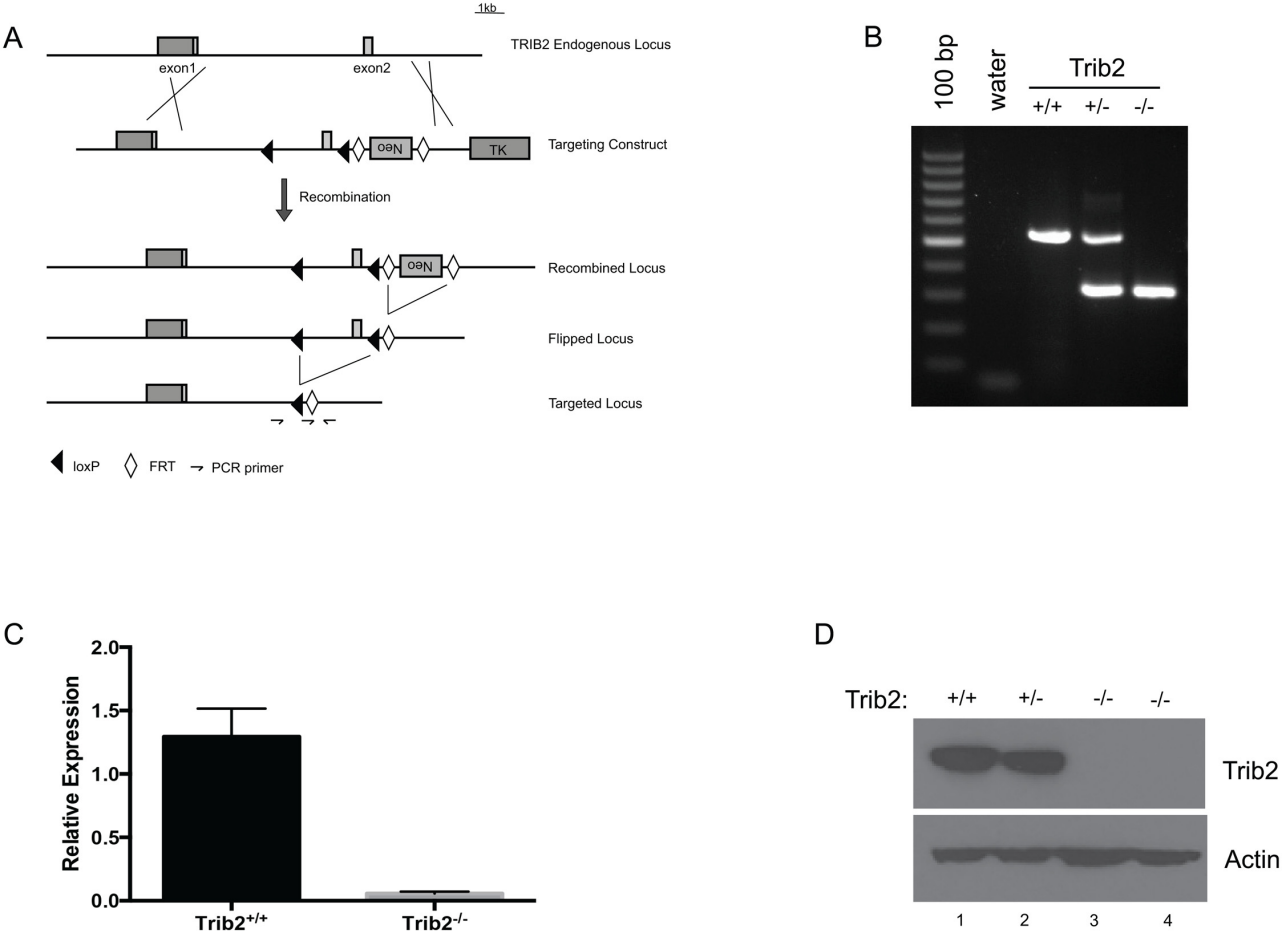
Generation and characterization of Trib2 null mice. A) The targeting strategy to delete *Trib2* exon 2 is shown. B) The deletion of the targeted region is shown by PCR performed on tail genomic DNA. C) qPCR; and D) immunoblot showing the deletion of Trib2 mRNA and protein in mouse thymocytes. Error bars indicate standard deviation.

Trib2 is highly expressed in developing murine T cells and murine and human T-ALLs [18]. We therefore generated a genomic Trib2 knockout mouse to determine the importance of Trib2 in T cell development and disease initiation. LoxP sites flanking exon 2 of Trib2 were inserted into a targeting vector and germline transmission was achieved through homologous recombination. The mice were subsequently crossed to FLP1-transgenic mice to remove the neomycin cassette and then backcrossed for greater than 8 generations to the C57BL/6 background (Fig 2A). Mice were born at the expected Mendelian frequencies without gross developmental anomalies (data not shown). Adult mice did not develop pathology or shortened survival (data not shown). Deletion of exon 2 of Trib2 was confirmed by PCR genotyping of genomic DNA from offspring (Fig 2B). Trib2 mRNA and protein expression was not detected in Trib2^-/-^ thymocytes as compared to Trib2^+/+^ or Trib2^+/-^ littermate controls (Fig 2C and 2D).

**Fig 3:**
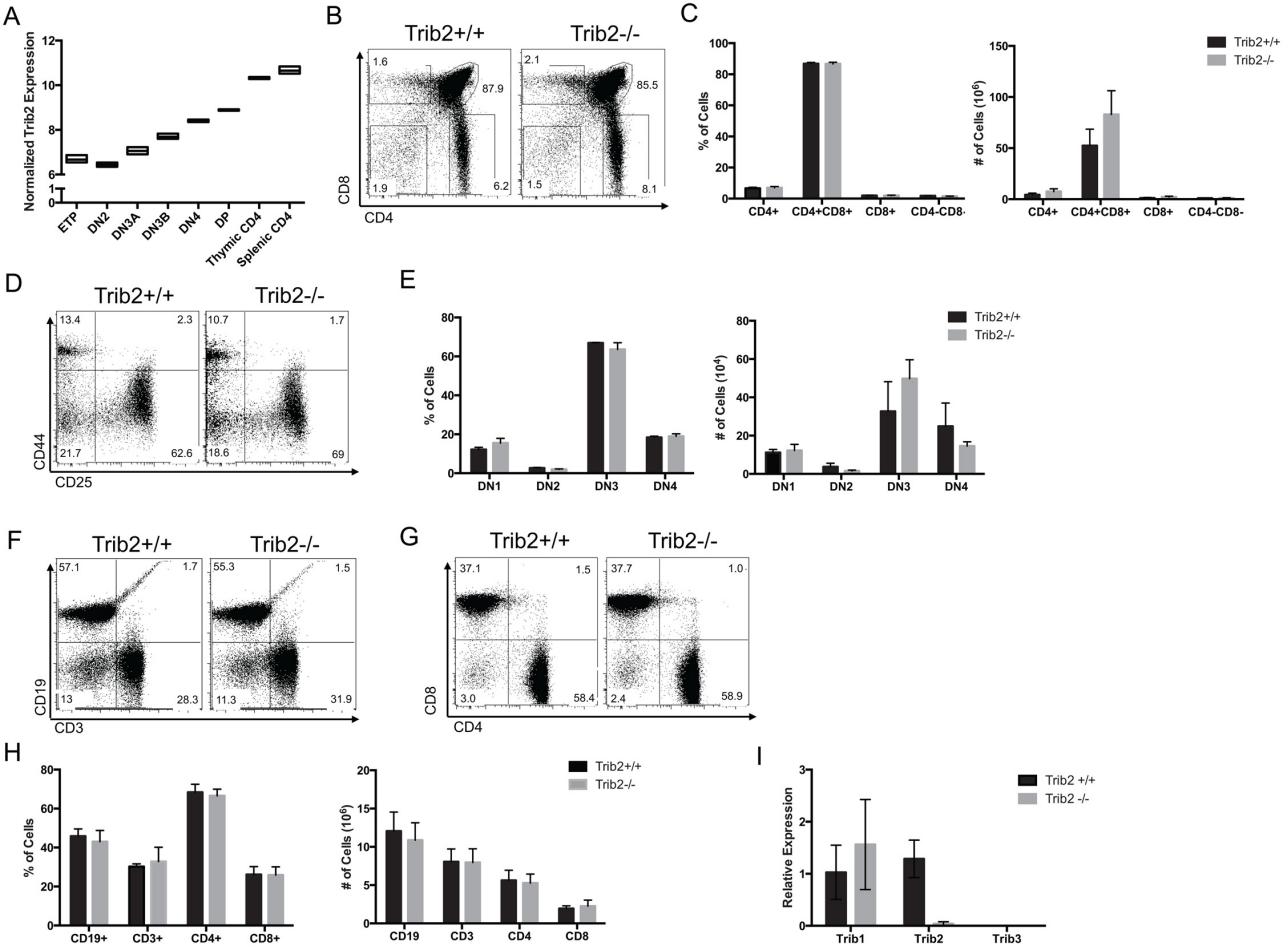
Deletion of Trib2 does not affect T cell development. A) Levels of Trib2 expression throughout T cell development as determined in the Immgen microarray data set are shown. B) Thymic subsets were analyzed by flow cytometry for CD4 and CD8 expression. Representative scatter plots (B) and C) graphs depicting the absolute numbers and percentages of cells in the total population are shown (n = 5–6 mice per group). D) CD4^−^CD8^−^ double negative thymocytes were further assessed for the surface expression of CD44 and CD25. Representative scatter plots and E) graphs depicting the percentages and absolute numbers of cells in the total population are shown (n = 3 mice per group). F) The surface expression of CD3 and CD19, and G) CD4 and CD8 on splenocytes was assessed (n = 6 mice per group). H) Representative scatter plots and graphs depicting the percentages and absolute numbers of cells in the total splenocyte population are shown (n = 6 mice per group). Error bars indicate standard deviation. I) The expression of Trib1 and Trib3 were measured by qPCR (n = 3 per group).

*Trib2* mRNA expression increases throughout T cell differentiation from T lineage precursors to mature, resting T cells (Fig 3A). We therefore analyzed the effect of Trib2 loss in both developing T cells and early hematopoietic progenitor populations in the bone marrow. Deletion of Trib2 did not affect the percentage of hematopoietic stem cells or progenitors in the bone marrow (S1A and S1B Fig). Additionally, the survival of Trib2^-/-^ mice did not differ from Trib2^+/+^ littermates in response to serial 5-fluorouracil treatment, suggesting an equivalent response to stress (S1C Fig). Immunophenotyping by flow cytometry showed no differences in the percentage or absolute numbers of T-lineage committed thymocytes (Fig 3B and 3C). Furthermore, no difference in the percentage or numbers of CD4/CD8 double negative cells at each stage of development was observed (Fig 3D and 3E). Additional data showed no difference in the percentage or numbers of CD19^+^ B cells or CD3^+^ T cells in the spleen (Fig 3F and 3H) or peripheral blood (data not shown) of Trib2^-/-^ mice. Finally, no difference in the percentage or numbers of CD4 or CD8-expressing T cells was observed in the spleens of mice lacking Trib2 (Fig 3G and 3H). In addition, there did not appear to be compensatory changes in either *Trib1* or *Trib3* expression in Trib2-deficient thymocytes (Fig 3I). Thus, Trib2 absence does not impair T cell development.

### Trib2 absence decreases latency and increases penetrance of Notch-induced T-ALL

**Fig 4:**
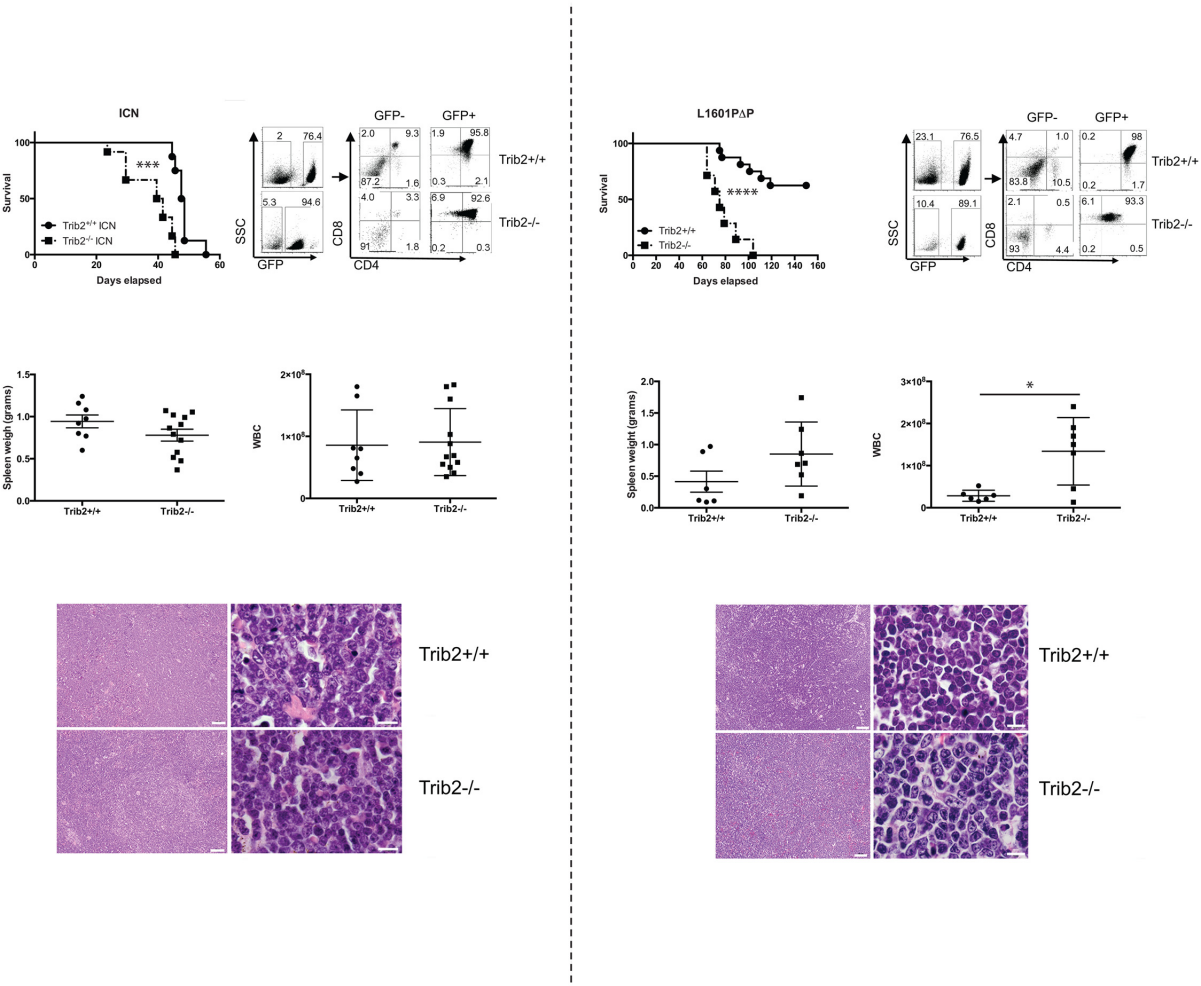
Loss of Trib2 decreases disease latency of Notch-driven T-ALL. A) Kaplan-Meier curve of lethally irradiated mice reconstituted with Trib2^+/+^ or Trib2^-/-^ cells expressing intracellular Notch1 (ICN; “***”, p = 0.0002). Mice with a body condition score of ≤2 and decreased mobility were euthanized (n = 8 Trib2^+/+^ and n = 12 Trib2^-/-^ recipients. B) Flow cytometry was used to assess the immunophenotype of leukemic cells in the spleen. C) Spleen weights and D) WBC of moribund mice are shown. E) Representative H&E staining of spleens harvested from moribund mice (left panels, 10X magnification, scale bar = 50μM; right panels, 100X magnification, scale bar = 10μM). F) Kaplan-Meier curve of lethally irradiated mice reconstituted with Trib2^+/+^ or Trib2^-/-^ cells expressing the weakly oncogenic Notch mutant, Notch 1 L1601PΔP (“****”, p<0.0001), (n = 16 Trib2^+/+^ and n = 7 Trib2^-/-^ recipients). G) Flow cytometry was used to assess the immunophenotype of leukemic cells in the spleen. H) Spleen weights and I) WBC of moribund mice are shown (“*”, p = 0.013). J) H&E staining of spleens from moribund mice (left panels, 10X magnification, scale bar = 50μM; right panels, 100X magnification, scale bar = 10μM).

As Trib2 absence did not affect steady-state T cell development, we employed a murine T-ALL model to determine the importance of Trib2 in disease pathogenesis. Hematopoietic stem and progenitor cells from either Trib2^-/-^ or wild-type (Trib2^+/+^) controls were transduced with retroviral supernatants expressing oncogenic forms of Notch along with GFP as a surrogate marker [24]. The cells were then injected into lethally irradiated syngeneic C57BL/6 recipients. Two different forms of oncogenic Notch were used for these studies; Notch1 L1601PΔP expresses a full length Notch1 allele that contains a destabilizing mutation in the HD domain and lacks the regulatory PEST domain, whereas ICN1 (intracellular Notch1) encodes only the intracellular Notch1 domain [24]. Notch1 L1601PΔP exhibits weaker activity than ICN1 in Notch1 reporter assays and induces T-ALL with longer latency and reduced penetrance [24]. Mice receiving Trib2^-/-^ cells expressing ICN1 succumbed to T-ALL more quickly than their wild-type counterparts, with a median survival of 40 and 47.5 days, respectively (Fig 4A). Despite a decrease in survival time, the absence of Trib2 did not affect the expression of CD4 and CD8 on the surface of leukemic cells (Fig 4B). Additionally, moribund recipient mice showed similar spleen weights (Fig 4C), white blood cell counts (WBC; Fig 4D) and immature blast morphology (Fig 4E). The leukemic cells filled the spleen, obliterating the normal splenic architecture (Fig 4E). Leukemic cells from both Trib2^-/-^ and Trib2^+/+^ donors also filled the bone marrow and infiltrated non-hematopoietic organs, such as liver and lung (data not shown). Consistent with previous results [24], L1601PΔP induced a partially penetrant phenotype with approximately 40% of the mice receiving Trib2^+/+^ cells transduced with L1601PΔP succumbing to T-ALL with a median survival of 97 days. In contrast, all of the mice receiving Trib2^-/-^ cells transduced with L1601PΔP developed leukemia with a median survival of 75 days (Fig 4F). When comparing the mice that developed leukemia induced by L1601PΔP, no difference was observed between the Trib2^+/+^ and the Trib2^-/-^ mice with regards to CD4 and CD8 expression on the leukemic cells (Fig 4G) or spleen weights (Fig 4H). Moribund Trib2^-/-^ recipients had significantly higher WBCs than the control group (Fig 4I). The immature blast histopathology of these T-cell leukemias was similar to that previously reported [24] and between the two groups (Fig 4J and not shown). The leukemic cells were capable of serial transfer; however, mice receiving sorted Trib2^-/-^ cells expressing either ICN or L1601PΔP succumbed to leukemia more quickly than those receiving sorted Trib2^+/+^ cells (S3 Fig). Thus, the absence of Trib2 increases the penetrance and decreases the latency of Notch1-induced T-ALL.

### Absence of Trib2 is associated with increased C/EBPα expression in T-ALL cells

**Fig 5:**
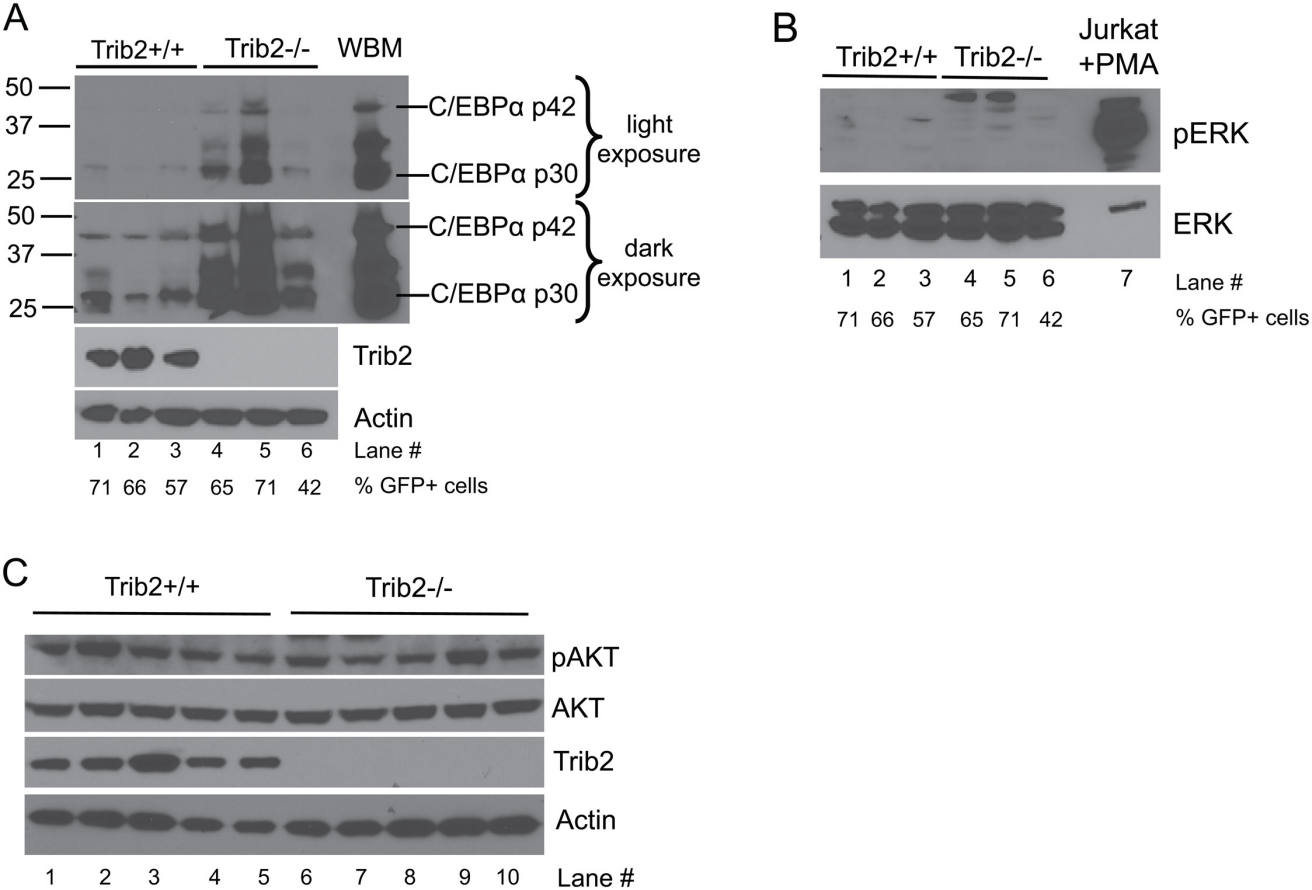
C/EBPα expression increases in the absence of Trib2 in primary tumor cells expressing oncogenic Notch. Immunoblotting was used to visualize A) the expression of C/EBPα, and B) the phosphorylation status of ERK in Trib2^+/+^ or Trib2^-/-^ splenocytes of moribund mice. Percentage of GFP^+^ cells in each sample; Lane 1: 71, Lane 2: 66, Lane 3: 57, Lane 4: 65, Lane 5: 71, Lane 6: 42. Whole bone marrow (WBM) (A) or Jurkat cells stimulated with PMA (B) were used as controls. In Panel A, lighter and darker exposures are provided. C) The phosphorylation status of AKT (pS473 AKT) was determined by immunoblot in GFP^+^ sorted splenocytes from moribund mice.

Trib2 regulates multiple signaling pathways including AKT [14, 16], MAPK [15] and C/EBPα [6]. There are two C/EBPα isoforms, p42 and p30, that are derived from post-transcriptional processing of a single transcript [25, 26]. C/EBPα p42 is anti-proliferative, whereas p30 is proliferative and a decrease in the p42/p30 ratio is associated with tumorigenesis [27, 28]. In myeloid cells, retroviral Trib2 expression decreased the p42/p30 ratio [6]. In the Trib2-deficient T-ALL cells, C/EBPα was markedly increased. Both the p42 and p30 isoforms were increased; however, p30 remained greater than p42 (Fig 5A). Despite a substantial increase in protein levels, the C/EBPα target genes *Mpo* [29], *Ltf* [30], and *Lyz2* [31] remained undetectable in sorted tumor cells from Trib2^+/+^ and Trib2^-/-^ mice, and expression of the myeloid marker *Itgam* did not change (data not shown). Phosphorylated ERK was not detectable in either group (Fig 5B) and no change in AKT phosphorylation was observed between Trib2^-/-^ and Trib2^+/+^ tumor cells expressing ICN1 (Fig 5C). Together, these data implicate Trib2 as a suppressor of disease initiation in Notch-driven T-ALL through a process that may involve C/EBPα.

### Absence of Trib2 does not affect AML induction by MLL-AF9

**Fig 6:**
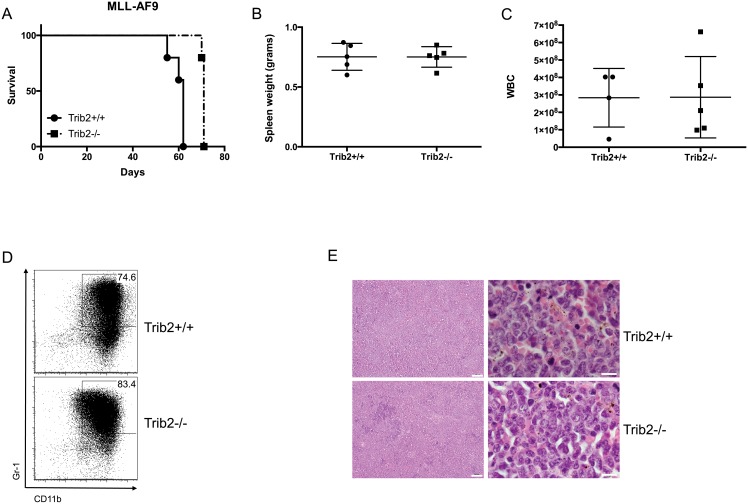
Trib2 does not suppress the initiation of MLL-AF9-driven myeloid leukemia. A) Kaplan-Meier curve of lethally irradiated mice reconstituted with Trib2^+/+^ or Trib2^-/-^ cells expressing MLL-AF9. Mice with a body condition score of ≤2 and decreased mobility were euthanized (n = 5 per group). B) Spleen weights and C) WBC of moribund mice are shown. D) Flow cytometry was used to assess the immunophenotype of leukemic cells in the spleen. E) H&E staining of spleens harvested from moribund mice (left panels, 10X magnification, scale bar = 50μM; right panels, 100X magnification, scale bar = 10μM).

One potential explanation for the decreased latency of Notch-induced T-ALL in the Trib2-null cells was that the hematopoietic progenitors were more susceptible to retroviral transduction. To address this possibility, we used the MLL-AF9 retroviral model of AML induction [32]. We used identical methods to those used for the Notch bone marrow experiments with the exception that the MigR1 retrovirus expressed MLL-AF9. In contrast to Notch-induced T-ALL, absence of Trib2 did not decrease disease latency in this model. Instead, there was a slight increase in disease latency with a median survival of 62 days or 71 days for recipients of Trib2^+/+^ or Trib2^-/-^ cells, respectively (Fig 6A). Both groups showed splenomegaly (Fig 6B) and increased WBCs (Fig 6C), while the animals that received empty vector were not affected (S2 Fig). The cells were largely Gr1^+^CD11b^+^ immature myeloid blasts that infiltrated multiple organs and filled the bone marrow (Fig 6D and 6E and data not shown). Thus, the decreased latency observed in the Notch T-ALL model is specific to T-ALL and not a general feature of leukemia induction in retroviral models transducing Trib2-null donor cells.

### Trib2 expression correlates with different T-ALL subtypes

**Fig 7:**
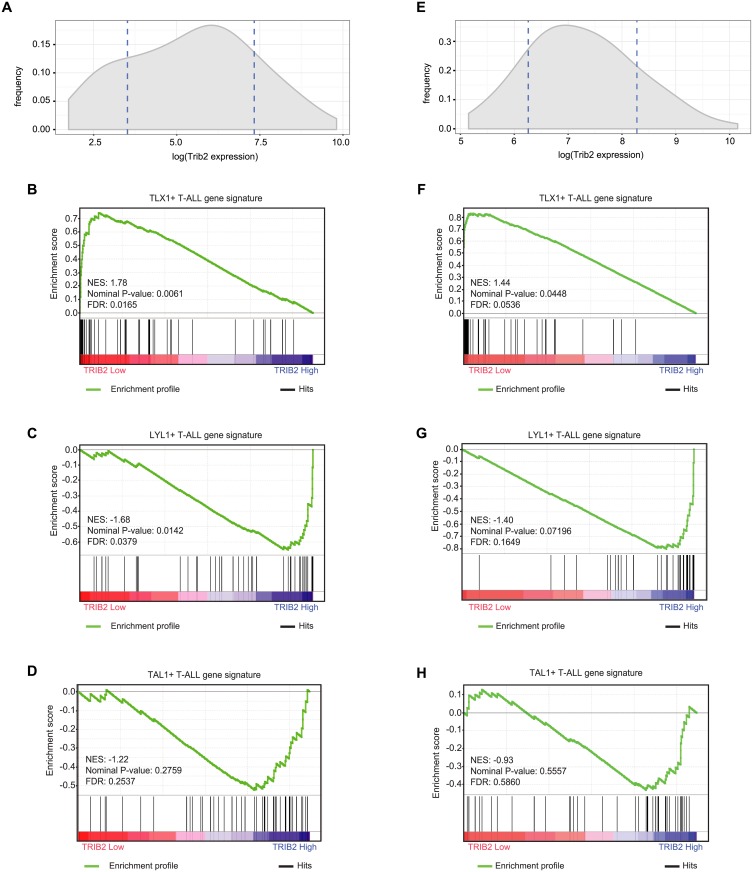
TRIB2 expression correlates with the gene signatures of human early immature and cortical mature T-ALL subtypes. A) Adult T-ALL samples with low (mean-SD) and high (mean+SD) *TRIB2* gene expression (average of 202478_at and 202470_s_at) were identified in the GSE13159 dataset. Dashed lines indicate mean-SD and mean+SD of TRIB2 gene expression. B, C) GSEA analysis shows enrichment of gene signatures of the TLX1^+^ (B) and LYL1^+^ (C), but not TAL1^+^ (D), adult T-ALL molecular subtypes in samples with low and high TRIB2 expression, respectively (FDR < 0.05). E) Pediatric T-ALL samples with low (mean-SD and high (mean+SD) *TRIB2* gene expression (average of 202478_at and 202470_s_at) were identified in the GSE26713 dataset. Dashed lines indicate mean-SD and mean+SD of TRIB2 gene expression. F, G) GSEA analysis shows enrichment of gene signatures of the TLX1^+^ (F) and LYL1^+^ (G), but not TAL1^+^ (H), pediatric T-ALL molecular subtypes in samples with low and high TRIB2 expression, respectively (FDR < 0.05). NES, normalized enrichment score.

Our finding that absence of Trib2 promoted Notch-induced T-ALL was surprising as *TRIB2* expression correlates with activated *NOTCH1* in primary patient tumor samples [18]. T-ALL is not a uniform disease; instead, molecular profiling revealed signatures that specify different subtypes. Primary samples from two separate datasets (GSE13159 and GSE26713 including adult and pediatric T-ALL samples, respectively) were identified based on the expression of the oncogenes TLX1, LYL1 and TAL1, and grouped based on unique expression profiles, which correlate with recognized stages of normal thymocyte development; LYL1+ samples resemble thymocytes in the pre-T double negative stage of development, while TLX1^+^ and TAL1^+^ samples resemble thymocytes at the early and late cortical stages, respectively [33]. To determine whether TRIB2 expression varied between subtypes, we correlated the level of *TRIB2* expression with the gene signatures of T-ALL molecular subtypes. The Gene Set Enrichment Analysis (GSEA) [34] analysis revealed a marked enrichment of the TLX1^+^ gene signature (cortical mature T-ALL) in low TRIB2 expressing T-ALL patients (Fig 7A, 7B, 7E and 7F), whereas the gene signature of LYL1^+^ (early immature T-ALL) significantly associated with samples expressing high levels of TRIB2 (Fig 7A, 7C, 7E and 7G). Interestingly, the gene signature of the TAL1^+^ T-ALL subtype (late cortical T-ALL) did not significantly correlate with the TRIB2 expression status (Fig 7D and 7H). Of particular significance, these correlations held true across two independent cohorts of T-ALL patients, including 174 adult (Fig 7A–7D) and 117 pediatric samples (Fig 7E–7H), respectively [35, 36]. Thus, these data suggest that Trib2 expression varies across human T-ALL subtypes.

## Discussion

Our data show an important role for Trib2 in the induction of T-ALL by oncogenic Notch. While Trib2 is not required to maintain either a Notch-induced or a Tal1-induced murine T-ALL cell line, disease latency was decreased in mice transplanted with Trib2^-/-^ cells expressing both strong and weak oncogenic Notch1 alleles. The weak oncogenic Notch1 allele L1601PΔP induced T-ALL in ~40% of mice with increased survival time relative to ICN1 when expressed in wild-type BM progenitors. However, when expressed in Trib2-deficient progenitors, penetrance increased to 100% while survival decreased compared to the wild-type controls. Together, these data suggest that Trib2 suppresses T-ALL pathogenesis. One possible explanation for our data is that the hematopoietic progenitors lacking Trib2 are more susceptible to retroviral transduction. If so, we would predict that leukemia survival would be reduced regardless of the oncogenic driver. This does not seem to be the case, however, as transduction of Trib2-deficient cells with the myeloid oncogene, MLL-AF9, did not decrease leukemia latency. This suggests that the decrease in leukemia survival is not a general property of Trib2-deficient hematopoietic progenitors.

In contrast to the effects of Trib2 absence on leukemogenesis, we did not observe effects on thymocyte development. This was somewhat surprising as the amount of active Notch1 in DN3 cells is similar to the amount in T-ALL cells [37], and *Trib2* is relatively highly expressed (at least compared to Trib1 and Trib3) during T cell development. Of particular relevance to our study, defects were not observed in the DN3 to DP populations that are suggested to contain the leukemic stem cells in different models of Notch1-induced T-ALL [4, 38–40]. In addition to the thymus, the splenic CD4^+^ and CD8^+^ populations were present in the expected ratios; however, we cannot rule out the possibility that T cell function may be perturbed in the absence of Trib2.

Given the relatively high expression of Trib2 in developing T cells and its positive correlation with human T-ALL [18], we were surprised to find that Trib2 functioned as a tumor suppressor in our murine Notch1-induced T-ALL model. This led us to investigate Trib2 expression in the different subtypes of human T-ALL. The results from two independent T-ALL cohorts showed that *TRIB2* was negatively correlated with the TLX1^+^ gene signature (cortical mature T-ALL) and positively correlated with the LYL1^+^ gene signature (early immature T-ALL). Interestingly, many of the genes associated with the expression of *TLX1* are involved in cell growth and proliferation, including *MYC* [33]. Additional work is required to understand the implications of these associations.

Although weak activating Notch1 mutations including L1601PΔP are frequent in human T-ALL [2], few cooperating events that result in increased disease penetrance *in vivo* have been identified. Similar to our data showing that the loss of Trib2 results in disease penetrance of 100%, the absence of the tumor suppressor PTEN [41] or expression of oncogenic KRAS [24] both increase penetrance and decrease the latency of T-ALL induced by L1601PΔP. It remains to be determined whether the comparable effects on L1601PΔP-mediated disease are due to similar mechanisms by these different perturbations.

Previous studies suggest that Trib proteins can function as both dominant oncogenes [6, 42, 43] and tumor suppressors [44]. Trib3 was shown to enhance the tumorigenicity of a variety of tumor cell lines and xenografts via a mechanism that involved dysregulation of AKT phosphorylation by the mTORC2 complex and subsequent inactivation of the transcription factor FOXO3 [44]. Trib1 and Trib2 are also implicated as tumor suppressors in some subtypes of AML through inhibition of JNK signaling [45]. In contrast, Trib2 transformation in a murine AML model depends on C/EBPα degradation by a COP1-dependent mechanism. The overexpression of Trib2 in AML does not result in total C/EBPα degradation in the tumor cells; instead, there appears to be preferential degradation of the p42 C/EBPα isoform that results in a relative increase in expression of the p30 C/EBPα isoform [6]. The two C/EBPα isoforms are generated by post-transcriptional processes [25, 26] and differ in their amino termini; the N-terminal region unique to p42 contains transactivation elements that interact with transcriptional machinery. The two isoforms share a common C-terminal region that interacts with the chromatin remodeling complex and other transcription factors [46]. Functionally, C/EBPα p42 is anti-proliferative via its suppression of E2F. Conversely, C/EBPα p30 is unable to interact with E2F resulting in unchecked proliferation [27]. Expression of C/EBPα p30 in the absence of p42 is sufficient to induce AML in mice through a mechanism involving loss of proliferative control in myeloid progenitor populations [28]. In developing T cells, C/EBPα transcription is repressed by the Notch target, Hes1, which is important for T cell specification [47], a mechanism that may be reinforced by Trib2-induced degradation of C/EBPα protein. Unlike the scenario in Trib2-induced AML, the p42:p30 C/EBPα ratio is maintained in the Trib2-deficient leukemic T-ALL cells; however, the major distinction is that there is much more total C/EBPα protein in the absence of Trib2. One potential explanation is that the increased amount of C/EBPα promotes the pro-proliferative effects of the p30 isoform. Nevertheless, how the increased levels of C/EBPα promote the more aggressive disease in our Notch1-induced T-ALL models remains to be determined.

In summary, we show that Trib2 absence does not have gross effects on mouse development, and in particular on T cell development. This suggests that in some contexts, therapeutically targeting Trib2 may be a well-tolerated treatment strategy. However, our studies also describe a new role for Trib2 in the pathogenesis of T-ALL by a mechanism that may involve increased C/EBPα expression. Although our data clearly show that Trib2 absence enhances the severity of Notch1-induced murine T-ALL, additional studies are required to determine the relevance of these findings to human T-ALL.

## Materials and Methods

### Animals

To generate the Trib2 null mice, a 129S7/SvEv BAC clone, which covers the entire TRIB2 genomic locus, was obtained from the Sanger Institute (http://www.sanger.ac.uk/). The BAC recombineering system was utilized to construct a targeting vector (http://recombineering.ncifcrf.gov/) [48]. Two loxP sites were inserted to sandwich exon2. The FRT-flanked Neomycin cassette was cloned downstream of the 3’ loxP site. TL1 ES cells were electroporated with the linearized targeting vector. Homologous recombinants were screened with a combination of PCR and Southern blotting. Once germline transmission was observed, the mice were mated with FLP1-transgenic mice (B6;SJL-Tg(ACTFLPe)9205Dym/J, The Jackson Laboratory) to remove the Neomycin cassette. The flippase gene was bred out in the subsequent matings. The mice were then backcrossed to the C57BL/6 background for more than 8 generations. All mice were housed in specific pathogen-free facilities at the University of Pennsylvania. Experiments were performed according to the guidelines from the National Institutes of Health with approved protocols from the University of Pennsylvania Animal Care and Use Committee. The Body Condition Score Index (BCSI) [49] was an important criterion for monitoring the health of the mice. Mice were monitored at least three times per week and euthanized by CO_2_ inhalation followed by cervical dislocation.

### Bone marrow transduction and transplantation

Bone marrow cells were collected from 6- to 12-week-old mice 4 days after intravenous administration of fluorouracil (5-FU) (250 mg/kg). The cells were cultured overnight in the presence of IL-3 (6 ng/ml), IL-6 (5 ng/ml), and SCF (100 ng/ml). The cells were then washed, resuspended in media containing cytokines and 4 μg/mL hexadimethidrine bromide (Sigma). Retroviral supernatants, normalized by titer, were added and the cells were centrifuged at 1,290 *g* for 90 minutes. A second round of spinoculation was performed the following day. After washing with PBS, at least 5 × 10^5^ cells were injected intravenously into lethally irradiated (9 Gy) recipients. For secondary recipients, 2x10^6^ sorted, GFP^+^ splenocytes were injected into sublethally irradiated (5 Gy) recipients. Mice were maintained on antibiotics in the drinking water for 2 weeks after BMT. Mice were bled every 2 weeks to monitor blood counts and evaluate the presence of circulating immature T cell progenitors by flow cytometry. Mice with a body condition score of ≤2 [49] and decreased mobility were euthanized and tissues were harvested for analysis.

### Serial fluorouracil treatment

Mice received an intravenous dose of 5-FU on Day 0 (250mg/kg), Day 7 (150mg/kg) and Day 14 (150mg/kg). Mice with a body condition score of ≤2 and decreased mobility were euthanized.

### Constructs and retroviruses

Production of high-titer virus was performed as described previously [50, 51]. Lentiviral pLKO.1 shRNA constructs were co-transfected into 293T/17 cells (ATCC, CRL-11268) with pMDL (gag-pol), pRSV-Rev and pHIT123 (envelope). Retroviral constructs were co-transfected into 293T cells with pCGP (gag-pol) and pHIT123 (envelope). Viral titers were determined using 3T3 fibroblasts. Viral supernatants were stored at -80°C.

### Cell culture and transduction

All cell lines were maintained in RPMI 1640 (Cellgro) supplemented with 10% fetal bovine serum (FBS) (Hyclone), 1% penicillin/streptomycin (Gibco), 1% L-glutamine (Gibco) and 100 μM 2-mercaptoethanol (Sigma). Cells were centrifuged with viral supernatant and 8 μg/mL hexadimethidrine bromide (Sigma) at 1,290 *g* for 90 min at 25°C. The cells were analyzed or sorted at the indicated times after transduction. For growth curves, 5x10^4^ cells were seeded. Cells were counted over time. Cell density was maintained at 1x10^6^ cells/ml.

### Flow cytometry and cell sorting

For the analysis of primary mouse cells, bone marrow, blood thymocytes and splenocytes were harvested and resuspended in PBS supplemented with 2% heat-inactivated FBS (Gibco). Antibodies used for cell surface staining (all obtained from eBiosciences except where noted) were CD3 (145-2C11), CD19 (1D3), CD4 (RM4-5), CD8 (53–6.7), CD25 (PC61; Biolegend), CD44 (IM7), Thy1.2 (53–2.1), CD11b (M1/70) and Gr-1 (RB6-8C5). Dead cells were excluded from analyses with 4',6-diamidino-2-phenylindole (DAPI). To detect Annexin V, Jurkat cells were stained following the manufacturer’s protocol. Briefly, an equal number of cells were resuspended in Annexin V binding buffer (10 mM Hepes pH 7.4, 140 mM NaCl, 2.5 mM CaCl_2_) with Annexin V antibody and incubated at room temperature in the dark for 15 minutes. The cells were then stored on ice and analyzed immediately. Cells were run on an LSR II flow cytometer (BD) and analyzed with FlowJo software v9.7 (TreeStar).

For cell sorting, primary splenocytes or cell lines were resuspended in PBS supplemented with 2% FBS, 1% penicillin/streptomycin and DAPI (for the exclusion of dead cells). GFP^+^ cells were sorted on a BD FACS Aria II using a 70μm nozzle at 70psi.

### Quantitative PCR

RNA was extracted using TRIzol. cDNA was synthesized from RNA with the SuperScript III kit (Invitrogen). Transcripts were amplified with Taqman PCR master mix (Applied Biosystems), and qPCR was performed on the ABI ViiA 7 system (Applied Biosystems). mRNA quantities were normalized to 18S. Primer/probe sets to Trib2 (Mm01270457_m1) and 18S (4319413E) were purchased from Life Technologies.

### Immunoblotting

Whole-cell lysates were prepared with RIPA buffer supplemented with protease inhibitors (Roche), NaF (10mM), PMSF (1mM) and Na_3_VO_4_ (1mM) or cells were directly lysed in SDS sample buffer. Proteins were separated using SDS-PAGE and transferred to PVDF membranes. Antibodies used for immunoblot from Cell Signaling Technology were pS473 AKT (4051), AKT (4691), pT202/Y204 ERK (9101), ERK (9102), Trib2 (13533), and C/EBPα (8178). The antibody against TAL1 was purchased from Santa Cruz Biotechnology (sc-12984). β-actin (A5316; Sigma) was used as a loading control. Secondary anti-mouse-HRP (NA931V) and anti-rabbit-HRP (NA934V) were obtained from GE Healthcare. Blots were visualized with SuperSignal west pico chemilumenscence (34080; Thermo Scientific) or SuperSignal west femto chemilumenscence substrate (34095; Thermo Scientific).

### Histology

Freshly dissected organs were fixed in 10% neutral buffered formalin, paraffin embedded, sectioned, and stained with hematoxylin and eosin. Images were captured using an Olympus BX41 or a Nikon Eclipse Ni microscope.

### Microarray data analysis

CEL files from GSE15907, Immunological Genome Project data Phase 1 [52], were normalized using robust multichip average (RMA) with the Bioconductor [53] “affy” package [54]. The normalized expression values of Trib2 in different T cell subsets were plotted in GraphPad Prism. Variation between biological replicates is indicated using a box and whisker plot.

### GSEA analysis

The microarray-based gene expression measurements of human T-ALL samples were obtained from the GSE13159 and GSE26713 datasets. The samples from GSE13159 were GCRMA background adjusted, quantile normalized, PM corrected and batch corrected [53]. The samples from GSE26713 were normalized using RMA. Based on the expression level of TRIB2 (average of TRIB2 probes 202478_at and 202470_s_at) the low and high expressing TRIB2 T-ALL groups were identified as the samples with less than mean-SD or higher than mean+SD TRIB2 expression, respectively. Standard GSEA analyses were performed using the gene sets defined in C2:CGP collection of MSigDB V5.1.

### Statistical analysis

All statistical analyses were performed using GraphPad Prism.

## Supporting Information

S1 Fig Loss of Trib2 does not affect hematopoietic stem cells. A) Flow cytometry was used to assess the presence of lineage^−^Kit^+^Sca-1^+^ (LSK) hematopoietic progenitors and lineage^−^Kit^+^Sca-1^+^CD150^+^CD48^−^ (LSK SLAM) hematopoietic stem cells in the bone marrow of Trib2^+/+^ and Trib2^-/-^ mice. Representative plots are shown B) The average percentage of LSK and LSK SLAM cells in the bone marrow of Trib2^+/+^ and Trib2^-/-^ mice is shown (n = 3 per group). C) Kaplan-Meier curve of mice serially injected with 5-FU. Mice received an intravenous dose on Day 0 (250mg/kg), Day 7 (150mg/kg) and Day 14 (150mg/kg). Mice with a body condition score of ≤2 and decreased mobility were euthanized (n = 5 per group; p = 0.06).
(EPS)

S2 Fig MigR1 does not affect hematopoietic reconstitution of lethally irradiated recipients. A) Flow cytometry was used to assess the immunophenotype of GFP^+^ cells in the spleen. B) The percentage of each cell type in the spleen is shown.
(EPS)

S3 Fig Kaplan-Meier curves of sublethally irradiated secondary recipients reconstituted with 2 x10^6^ sorted Trib2^+/+^ or Trib2^-/-^ tumor cells isolated from primary recipients expressing A) ICN1 (n = 15 per group; “*”, p = 0.0217), or B) L1601PΔP (n = 5 Trib2^+/+^ recipients, n = 10 Trib2^-/-^ recipients; “***”, p = 0.0005). (EPS)

S4 Fig Trib2 is a transcriptional target of Notch1 in TAL-130, but not Jurkat, cells. A) Jurkat, or B) TAL-130 cells were treated with 1μM of the GSI Compound E or DMSO for 48 hours and then the GSI was “washed out” and replaced with DMSO to permit Notch signaling. After 4 hrs, RNA was isolated and subjected to reverse transcription followed by quantitative PCR to determine the expression levels of Trib2 or Hes1 relative to EF1α.
(EPS)
